# Special aspects of systemic inflammation course in animals

**DOI:** 10.14202/vetworld.2019.932-937

**Published:** 2019-07-02

**Authors:** Svetlana Vladimirovna Chernigova, Yury Vladimirovich Chernigov, Yury Anatolyevich Vatnikov, Evgeny Vladimirovich Kulikov, Irina Anatolyevna Popova, Vyacheslav Ivanovich Shirmanov, Mariya Andreyevna Molchanova, Irina Fedorovna Likhacheva, Yuliya Yuryevna Voronina, Darya Mikhaylovna Lukina

**Affiliations:** 1 Department of Veterinary Medicine, Omsk State Agrarian University Named After P. A. Stolypin, Omsk, Russia; 2 Department of Livestock, Omsk Agricultural Research Center, Omsk, Russia; 3 Department of Veterinary Medicine, Peoples’ Friendship University of Russia (RUDN University), Moscow, Russia

**Keywords:** dogs, purine mononucleotides, surgical sepsis

## Abstract

**Aim:**

In this study, we identified characteristics of systemic inflammation associated with surgical sepsis in animals. We evaluated the role of purine metabolism, functionally associated lipoperoxidation processes of membrane structures, and the antioxidant system in the development of surgical sepsis in dogs.

**Materials and Methods:**

Dogs with a provisional exclusion of sepsis were included in the study. The control group (Group 1) included clinically healthy dogs (n=5), and medium-breed dogs with systemic inflammation response syndrome (n=30) were categorized in the experimental group (Group 2). Along with hemogram and biochemical analysis, we determined the amount of malondialdehyde, glutathione, superoxide dismutase, catalase, glutathione reductase, and glucose-6-phosphate dehydrogenase on the 1^st^ and 14^th^ day of the study. Treatment included a thorough reorganization of the septic focus, followed by antibacterial therapy. Sick animals were injected with a drug (dexamethasone) that suppresses the synthesis and inhibits the action of inflammatory mediators. Decompensation of the functions of organs and systems was carried out using symptomatic therapy.

**Results:**

We found that enhanced lipid peroxidation of unsaturated fatty acids of membrane structures stimulates the generalization of inflammatory process, as evidenced by the significant deviation from the physiologically normal values of lipid peroxidation, C-reactive protein, blood cell count, etc. The course of systemic inflammation associated with surgical sepsis in animals can be attributed to several consistently developing processes that function as a result of increased purine mononucleotide catabolism, peroxide compound formation, and their excessive breakdown in reactions associated with the consumption of glutathione due to the insufficient recovery of glutathione disulfide.

**Conclusion:**

The amount of uric acid, glycosaminoglycans, hyaluronic acid in blood plasma, and the content of malondialdehyde, glutathione, and glutathione reductase in erythrocytes should be considered when assessing the severity of the systemic inflammatory process. The increased glutathione requirement in dogs with surgical sepsis requires intervention with pharmacological agents, and further research is needed in this aspect.

## Introduction

Damage to cells, tissues, and organs during surgical sepsis are mainly caused due to active oxygen metabolites (AOMs), which are intermediate products of aerobic metabolism. The most common among these products, according to researchers, is the superoxide radical, which is formed by elimination of an electron from the outer orbital of the oxygen molecule (O_2_−), forming a singlet oxygen; this occurs due to transition of the outer electron layer to the excited state, the nitroxyl radical (NO) and hydrogen peroxide (H_2_O_2_). Interaction of superoxide radical with H_2_O_2_ in the presence of iron ions leads to the for mation of a hydroxyl radical (OH), which is one of the strongest known oxidizing agents and is able to directly provoke the peroxidation of cell membrane structures [1-3].

It should be noted that the predominant source of active superoxide radicals and H_2_O_2_ in cells is xanthine oxidase (XO), which is activated by high concentrations of hypoxanthine and xanthine the products of adenosine monophosphate (AMP) catabolism. This process is enhanced by the excessive disintegration of cells, accompanied by change in the structure of nucleic acids to AMP [4-6]. These processes are significantly accelerated due to generalized inflammation associated hypoxia, leading to a decrease in the adenosine triphosphate formation in the mitochondria [7,8]. The increased demand for glutathione in dogs with surgical sepsis dictates the importance of intervention with pharmacological agents and further research.

This study was conducted to identify the features of systemic inflammation in surgical sepsis in animals, and to study the role of purine metabolism, functionally associated lipoperoxidation processes of membrane structures, and the antioxidant system in the development of surgical sepsis in dogs.

## Materials and Methods

### Ethical approval

The present study was carried out in accordance with the State program of Peoples’ Friendship University of Russia (RUDN University). Selective research on animals was conducted after receiving approval from the Bioethics commission SREC PFUR.

### Study area

The studies were carried out within the framework of the implementation of the research topic AAAA-A16-116040610034-2 “Development of surgical tools and methods to improve the quality of life of animals and evaluate productive properties.” Dogs with a provisional exclusion of sepsis were studied.

### Experimental animal

Systemic inflammation response syndrome (SIRS) in animals was recorded on the basis of anamnesis, clinical examination, and the results of a complete blood count. Diagnosis of surgical sepsis was based on the presence of the source of infection and SIRS [9-11]. The number of diseased animals was 30 dogs of both sexes and medium-breeds (n=30, Group 2). The control group (Group 1) included clinically healthy dogs (n=5).

### Research design

Along with hemogram and biochemical analysis, we determined the amount of malondialdehyde, glutathione, superoxide dismutase, catalase, glutathione reductase, and glucose-6-phosphate dehydrogenase on the 1^st^ and 14^th^ day of the study.

### Treatment management

We utilized standard and most commonly used local and general treatment to address surgical sepsis. Treatment of the dogs in Group 2 included a thorough reorganization of the septic focus, followed by antibacterial therapy, consisting of a combination of two antibiotics amoxicillin and cephalosporin, and an infusion-transfusion therapy using a Infesol solution. Sick animals were injected with a drug (dexamethasone) that suppresses the synthesis and inhibits the action of inflammatory mediators Decompensation of organs and systems functions was carried out using symptomatic therapy. On the 1^st^ day of treatment, the drugs were administered in “shock” doses, and further dosing of the drugs was carried out depending on the clinical condition.

### Statistical analysis

The obtained data were processed by the method of variation statistics using the Statistica software package for Windows version 6.0 (Stat Soft Inc., USA).

## Results and Discussion

An inflammatory response develops to protect the body from either injury or infection. The key factors of systemic inflammation are pathogen and damaged cell areas. There are several discrete mediators and cells controlling inflammatory response. These include important molecules such as prostaglandins, cytokines, and leukocytes. Although infections lead to inflammation, not all inflammatory reactions are initiated by an infectious etiology. SIRS criteria can be used to determine systemic inflammation, which includes the measurements of heart rate, respiratory rate, and body temperature, as well as laboratory measurements of leukocytes. Corresponding systemic inflammatory response removes harmful or infectious agents that initiated the process and returns the body to a state of homeostasis. Classification of surgical sepsis and scoring clinical and neurological status, revealed that most often systemic inflammation in dogs was caused by abdominal nosology in the age group of 6-7 years old (Table-1).

**Table 1:** Clinical characteristics of dogs in the experimental Group 2 on the 1^st^ day of the study, n=30 (M±m).

No.	Nosological entity	Number of animals	Age	Body weight	Clinical and neurological status points
1	Obstetric-gynecologic surgery	9	7.22±0.57*	25.11±2.25	81.89±1.20*
2	Penetrating injury of peritoneum	10	6.40±0.52*	27.20±1.70*	86.60±1.75
3	Bone fracture	5	6.80±0.58*	27.40±2.36	84.60±1.99*
4	Enterotomy	6	6.83±0.31*	27.33±2.16	84.50±2.57
Half value			6.81±0.17*	26.76±0.55*	84.40±0.97*

* p<0.05

An examination of the blood of experimental animals of Group 2 on the 1^st^ day showed that they actively underwent nucleic acid destruction to form purine nucleotides: AMP and guanosine monophosphate (GMP), characterized by total destruction of damaged cells. This process is evidenced by an excess of plasma uric acid concentration in dogs of the second group (962.33±2.65 μmol/l) than the control indicator (619.13±5.21 μmol/l) by 55.43%. AMP and GMP are further catabolized to hypoxanthine, which is oxidized by XO to uric acid (Table-2).

**Table 2:** Changes in the level of lipid peroxidation in dogs on the 1^st^ day of the study, M±m.

Value	Unit	Group1, control, n=5	Group2, n=30
Uric acid	μmol/l	619.13±5.21	962.33±2.65
Superoxide dismutase	units/mL	277.90±1.63	270.53±0.62
Catalase	IU/l	62.2±0.46	80.1±0.45
Malondialdehyde	μmol/l	257.10±0.93	431.1±0.46
Glutathione	mol/l	1.04±0.02	1.54±0.04
Glutathione reductase	IU/l	0.02±0.00	0.09±0.00
Glucose-6-phosphate dehydrogenase	IU/l	1.54±0.01	1.95±0.03
Erythrocyte	×10^12^/l	6.6±0.72	3.23±0.14
Hemoglobin	g/dl	16.1±0.54	9.1±0.31
Hematocrit	%	41.7±1.11	25.8±1.02
ESR	mm/h	3±1.0	10.2±2.33
C-reactive protein	mg/l	4.2±1.37	11.7±15.77
Leukocytes	×10^9^/l	9.3±2.43	15.2±2.25

*p<0.05

Along with an increase in the AMP and GMP levels, these nucleotides are cleaved to form hypoxanthine, which in our opinion, is upregulated by hypoxia that is concomitant with the process of inflammation, leading to acidification of the tissue environment. Adenylate deaminase and adenosine deaminase activity involved in the catabolism of purine mononucleotides simultaneously shows a significant increase with decreasing pH [12-14]. As a result of a biochemical reaction catalyzed by XO, hypoxanthine gets oxidized to xanthine and uric acid and this is always associated with the production of superoxide radicals and H_2_O_2_ using this enzyme. Furthermore, due to this interaction, a hydroxyl radical is formed, which is one of the most powerful oxidizers in nature and is capable of oxidizing unsaturated fatty acids of phospholipids in cell membrane structures, leading to the destruction of the cells [15-17]. Subsequently, the hydroperoxides are broken into fragments (one of which is malonic dialdehyde) to fuse with thiobarbituric acid to form colored compounds. The amount of malondialdehyde in the erythrocytes of dogs of Group 2 was 431.1±0.46 μmol/l, which is 67.7% more than in the control group (257.10±0.93 μmol/l). A sharp increase in this indicator is associated with an effect on the erythrocyte membrane of AOMs, which is generated by XO during inflammation in the blood plasma [18-20].

The sharp increase in uric acid concentration in the blood plasma of animals and malondialdehyde levels in erythrocytes, is inextricably linked with an increase in catalase activity in these cells. Its value in sick animals is 80.1±0.45 IU/l, exceeding that in the control group by 28.78% (62.2±0.46 IU/l). Together, these indicators serve as a rationale for increased H_2_O_2_ production [21,22].

It can be stated that the compensatory mechanisms appearing in animals, to destroy H_2_O_2_ due to its increased formation. Considering that the XO and superoxide radical production is parallelly in parallel, one could also expect an increase in the superoxide dismutase activity [23-25]. However, the levels of this indicator in erythrocytes not only did not increase, but also showed a tendency to decrease by 2.65% than the control. In Group 2, the amount of superoxide dismutase was 270.53±0.62 units/mL and in Group 1 277.90±1.63 units/ml. We confer that this is the result of a direct impact on this AOM enzyme.

Superoxide dismutase activity can also be significantly inhibited by exploiting its insufficiently effective biosynthesis due to the deficiency of zinc and copper, which are necessary to form the superoxide dismutase active center. It should be noted that the lack of activation of this enzyme under enhanced AOM production conditions may lead to an unfavorable consequence for the cells. Superoxide dismutase itself is one of the most active enzymes in nature, is relatively insensitive to damaging effects, and it con tains a sulfhydryl group capable of oxidation [26-28].

The high levels of malonic dialdehyde and glutathione in erythrocytes is the evidence of enhanced peroxidation of membrane structures. Glutathione concentration in animals of Group 1 (1.04±0.02 mol/l) by 48.08%, while Group 2 shows 1.54±0.04 mol/l, this can be attributed to the manifestation of compensatory mechanisms in the organism.

Reduction of glutathione disulfide, formed during the inactivation of peroxide compounds, was inhibited in the diseased animals. This may be another evidence associated with the insufficient supply of glutathione to the tissues. The animal subsequently experiences a sharp deficit of nicotinamide adenine dinucleotide phosphate-H2 (NADP-H2), which is generated from glucose in the pentose cycle reactions.

Erythrocytes in dogs belonging to Group 2 demonstrated a 26.62% increase in glucose-6-phosphate dehydrogenase activity than the control (1.95±0.03 IU/l in the second group against 1.54±0.01 IU/l in the first), suggesting an increased NADP-H2 production due to the concomitantly increased tissue demand.

Decreased glutathione reductase activity leads to the activation of the reduction of glutathione disulfide in erythrocytes. On the 1^st^ day, the Group 2 showed an increase in glutathione reductase activity (0.09±0.00 IU/l), which was 4 times more than Group 1 (0.02±0.00 IU/l). This, in our opinion, is one of the factors leading to the deficiency of glutathione in the tissues.C-reactive protein is one of the proteins associated with the acute phase of inflammation, the concentration of which on the 1^st^ day in the blood plasma of Group 2 was increased by 115.2% than the control. The prevalence of tissue damage is directly proportional to the biosynthesis of this protein, and therefore, the level of the indicator [29,30].

Presence of systemic inflammation in animals on the 1^st^ day of the study can be confirmed with a sharp increase in the erythrocyte sedimentation rate (ESR) and the number of blood cells. ESR was increased by 240.8% than the control. In Group 2, ESR was 10.2±2.33 mm/h, and in Group 1 it was 3±1.0 mm/h. The leukocyte levels in sick dogs were also higher than in healthy dogs (15.2±2.25×10^9^/l in Group 2 and 9.3±2.43×10^9^/l in Group 1). An approximate increase of 69.7% in the number of leukocytes in the blood may indicate active phagocytosis in damaged tissues and organs. This increase occurs at the expense of other blood cells, such as neutrophils and monocytes. In Group 2, concentration of these cells in the blood exceeds that of animals in Group 1 by 78.1 and 291.7%, respectively. A slight increase (14.9%) in the lymphocyte number was recorded, which may serve as evidence that immune responses are activated in animals with systemic inflammation. Under the given conditions, it was noted that the number of eosinophils and basophils remains unchanged, attributing to the fact that these cells do not actively participate in the development of the body’s immune response and possess weak phagocytic activity.

Despite the traditional therapy for animals with systemic inflammation, after 14 days, the metabolic disorders described above differed significantly from the control indicators. Destructive changes in the tissues continued to prevail, evidenced by the increased concentration of uric acid (Table-3). In Group 2 the concentration of the blood plasma, by the end of the 2^nd^ week, was 25.12% higher than those the first group (774.63±3.75 μmol/l in the second group and 621.11±4.19 µmol/l in the first).

**Table 3:** Changes in the level of lipid peroxidation in dogs on the 14^th^ day of the study, M±m.

Value	Unit	Group1, control, n=5	Group2, n=30
Uric acid	μmol/l	621.11±4.19*	774.63±3.75*
Superoxide dismutase	units/mL	264.17±3.42*	262.40±1.06*
Catalase	IU/l	60.8±1.07*	77.63±0.50*
Malondialdehyde	μmol/l	249.5±1.34*	458.27±1.40*
Glutathione	mol/l	1.13±0.05*	1.31±0.02*
Glutathione reductase	IU/l	0.03±0.00	0.11±0.00
Glucose-6-phosphate dehydrogenase	IU/l	1.48±0.03*	1.98±0.03*
Erythrocyte	×10^12^/l	6.25±0.64	8.13±1.2
Hemoglobin	g/dl	17.7±0.73	14.9±2.4
Hematocrit	%	42.4±1.08	48.1±3.1
ESR	mm/h	3.3±0.9	6.0±1.7
C-reactive protein	mg/l	4.1±1.37	9.4±3.4
Leukocytes	×10^9^/l	8.1±1.81	14.8±2.6

* p<0.05

Development of lipid peroxidation processes in the erythrocytes of dogs in group 2 by the end of the 2^nd^ week is indicated by the indicators presented in Table-3. It is clear that the treatment of animals does not have a positive effect on the intensity of activated oxygen metabolite production. In the second group, catalase activity continues to significantly increase and amounts to 77.63±0.50 IU/l, with its concentration being 24.81% higher than that in the first group (60.8±1.07 IU/l), suggesting the continued enhanced production of H_2_O_2_ cells.

In Group 2, there was a decrease in the superoxide dismutase activity by 5.58% than that of the first group (262.40±1.06 in the second group and 264.17±3.42 in the first group), associated with inhibition of inactivation of superoxide radicals produced in parallel with H_2_O_2_.

Subsequently, increase in the malondialdehyde concentration in erythrocytes remained significantly increased compared with the control, by 78.25%, indicating preserved excessive lipid peroxidation of erythrocyte membrane structures in group 2. Enhanced production of peroxide compounds triggered the mechanisms of their destruction in the reactions associated with the oxidation of glutathione, demonstrated by an increase of 25.96% glutathione content in the erythrocytes of group 2 (1.31±0.02 mol/l) than that in the control group (1.13±0.05 mol/l), i.e., it is obvious that the this tripeptide remains elevated and 2 weeks after the start of treatment.

The lack of glutathione in tissues is a consequence of the insufficiently effective glutathione disulfide reduction, formed during its oxidation, requiring NADP-H2, As evidenced by an increase in erythrocyte activity of glucose-6-phosphate dehydrogenase in the second group (1.98±0.03 IU/l) by 28.57% than the animals of the control group (1.48±0.03 IU/l). Due to the increased transfer of glutathione to the damaged tissue structures from the liver and kidneys, it is our understanding that the body constantly experiences the need for its restored form. This requires NADP-H2, the formation of which depends on the glucose-6-phosphate dehydrogenase concentration. The increased formation of NADP-H2 is possible with the intensification of the reactions of the pentose cycle. Fourteen days after the start of treatment, we noted more than 5 times an increase in glutathione reductase activity in erythrocytes than the control (0.11±0.00 IU/l in the second group and 0.03±0.00 IU/l in the first), indicating decreased utilization of hydrogen ions carried by NADP-H2.

Generalized inflammation processes continued to increase in this period of observation, as demonstrated by a significant increase in the C-reactive protein concentrations in the blood plasma by 77.3% and ESR by 101.9% than those in the control animals. Thus, ESR values in animals of the second group was 6.0±1.7 mm/h, while in healthy animals was 3.3±0.9 mm/h. On the 14^th^ day of observation, the blood leukocyte count remained excessively high and exceeded similar benchmarks, being 14.8±2.6×10^9^/l in the second group, than 8.1±1.81×10^9^/l in the first. However, it should be noted that metabolic disorders in group 2 animals continued to stimulate hypoxia with a subsequent increase in the erythrocyte count by 25.0% compared with the control (8.13±1.2×10^12^/l in the second group and 6.25±0.64×10^12^/l in the first). Therefore, we can conclude that there is a tendency for hematocrit to increase (by 15.2%) with the decrease in the hemoglobin content in the blood (by 11.6%). In dogs of the second group, the hematocrit value was 48.1±3.1% and hemoglobin 14.9±2.4 g/dl; however, for those in the first group, the hematocrit value was 42.4±1.08% and hemoglobin 17.7±0.73 g/dl.

Thus, enhanced lipid peroxidation of unsaturated fatty acids of membrane structures stimulates the generalization of the inflammatory process, evidenced by a significant deviation from the physiological norm associated with lipid peroxidation, C-reactive protein, blood cell count, etc. We can consistently attribute the disease course of systemic inflammation in animals with surgical sepsis to developing processes that function as a result of increased purine mononucleotide catabolism, increased peroxide compounds formation and their excessive destruction in reactions associated with the expenditure of glutathione following the insufficient glutathione disulfide recovery.

## Conclusion

Effects of systemic inflammation were associated with successively developing processes in sepsis: excessive purine mononucleotide catabolism, intensification of the formation of peroxide compounds, and leading to the development of glutathione deficiency following the insufficiently effective glutathione disulfide recovery. When assessing the severity of the systemic inflammatory process, one should take into account the amount of uric acid, glycosaminoglycans, hyaluronic acid in blood plasma, and malondialdehyde, glutathione, and glutathione reductase in erythrocytes. The increased demand for glutathione in dogs with surgical sepsis dictates the importance of intervention with pharmacological agents.

## Authors’ Contributions

SVC and YVC planned, designed, and supervised the experiments; YAV and EVK carried out the research work; IAP, YYV, and DML super vised in the collection of sample and laboratory work; and VIS, MAM, and IFL supervised in drafting and revising the manuscript critically for important intellectual content**.** All authors read and approved the final manuscript.
